# Deep Multiview Image Fusion for Soybean Yield Estimation in Breeding Applications

**DOI:** 10.34133/2021/9846470

**Published:** 2021-06-23

**Authors:** Luis G. Riera, Matthew E. Carroll, Zhisheng Zhang, Johnathon M. Shook, Sambuddha Ghosal, Tianshuang Gao, Arti Singh, Sourabh Bhattacharya, Baskar Ganapathysubramanian, Asheesh K. Singh, Soumik Sarkar

**Affiliations:** ^1^Department of Mechanical Engineering, Iowa State University, Ames, Iowa, USA; ^2^Department of Agronomy, Iowa State University, Ames, Iowa, USA; ^3^Department of Computer Science, Iowa State University, Ames, Iowa, USA

## Abstract

Reliable seed yield estimation is an indispensable step in plant breeding programs geared towards cultivar development in major row crops. The objective of this study is to develop a machine learning (ML) approach adept at soybean (*Glycine max* L. (Merr.)) pod counting to enable genotype seed yield rank prediction from in-field video data collected by a ground robot. To meet this goal, we developed a multiview image-based yield estimation framework utilizing deep learning architectures. Plant images captured from different angles were fused to estimate the yield and subsequently to rank soybean genotypes for application in breeding decisions. We used data from controlled imaging environment in field, as well as from plant breeding test plots in field to demonstrate the efficacy of our framework via comparing performance with manual pod counting and yield estimation. Our results demonstrate the promise of ML models in making breeding decisions with significant reduction of time and human effort and opening new breeding method avenues to develop cultivars.

## 1. Introduction

Plant breeding programs worldwide rely on yield testing to make selections and advancement decisions towards the development of new varieties. A vital component in this process is the growing and harvesting of an inordinate number of plots at several locations each year, incurring substantial costs and resource allocations burdening the economics of a breeding program [1]. The need to assess tens of thousands of genotypes in a program is necessitated by the inherent requirement to work with a desired level of and expand the genetic variance for a higher response to selection [2]. Therefore, the need to accurately measure or predict yield has motivated researchers to constantly develop modern tools in genomics [3, 4] and phenomics [5–7] .

One of the avenues to yield prediction is through the fusion of high dimensional phenotypic trait data using machine learning (ML) approaches to provide plant breeders the tools to do in-season seed yield prediction [8] and fusing ML and optimization techniques to identify a suite of in-season phenotypic traits collected from multiple sensors that decrease the dependence on resource-intensive end-season phenotyping in breeding programs [9]. Other avenues have been through integrating weather and genetic information in conjunction with deep time series attention models for soybean seed yield prediction [10, 11].

These advances in ML methods and earnest effort to collect large data sets is commendable and has a positive role in numerous scenarios; however, these approaches do not work for plant breeding programs of all sizes, geographical regions, and crops. One less explored approach is using simple camera (triband digital red, green, blue (RGB)) to estimate plant reproductive organs and estimate seed yield. If imaging is coupled with automated ground robotic systems, breeders can compute plot seed yield to make breeding decisions in an efficient manner. Gao et al. [12] deployed a low cost lightweight distributed multiple robot system for soybean phenotypic data collection, which demonstrates a usable platform to meet yield estimation requirements. We envision that an automated data collection platform (i.e., ground robot) with sensors (i.e., digital camera) creates a framework to estimate seed yield in field conditions from breeding plots where genotypes are assessed for their merit and commercialization potential. The motivation for this challenge is to provide a timely and cost effective solution for seed yield estimation in field plots.

Computer vision models for crop yield estimation have been proposed in the past. However, such models are primarily built for larger fruit trees, with potentially less background clutter and occlusion compared to soybean plants and pods. For example, fruit harvesting robots [13], grape yield estimation in vineyard [14], fruit detection using thermal and RGB images [15], and apple fruit detection and yield estimation using RGB images [16, 17]. Recently, researchers proposed TasselNet [18] to count maize tassels based on a single aerial image in field conditions using computer visions methods. In sorghum, aerial imagery with deep learning was utilized for sorghum head counting [19]. However, these problems either worked on large plant organ (e.g., fruits) or minimal occlusion issues, which are present in crops with plant organs that overlap or are smaller sized. In particular, soybean pod counting with RGB image fusion from recorded frames is still an open research problem. In this context, we develop a deep multiview image fusion framework for soybean pod detection to predict the number of pods in each plot using multiple views of plants taken by simple RGB cameras. This method attempts to deal with the high level of occlusion that is caused by soybean plant architecture and predicts the total number of pods in a plot and not just what can be labeled by a human rater onscreen. Ground truth data for the total number of pods was done in this experiment by researchers meticulously hand counting every pod of each harvested plot. The need for multiple images to deal with occlusion and to generate more accurate estimates is supported by [20], where Hemming et al. found that fruit detection in peppers was significantly increased by adding multiple view points of the plant. We also test the usefulness of this model on images collected by a ground robot capable of taking RGB images that are processed by the deep learning model. While the proposed pod counting and yield estimation approach can be used in an offline manner using single or multiview images of plots, we further develop a plant detector and tracker (from soybean plots), enabling our approach to also be used in an online manner by a ground robot using on-board processors and edge computing. The rest of the paper is organized as follows.  Section 2 (Materials and Methods) presents the data collection and processing steps along with the ML framework for pod counting and plant detection/tracking from plots. In Section 3 (Results), we present the performance of our proposed framework by comparing performance with manual ground truth. In Section 4 (Discussion), we discuss the feasibility and promise of our approach in breeding programs. Finally, the paper is summarized and concluded with directions of future work.

## 2. Materials and Methods

In this section, we describe data collection and preprocessing steps essential for this project, followed by details on the deep learning framework for pod detection and counting, a framework for plant detection and tracking from soybean plots that can enable real-time pod counting (and yield estimation) using a ground robot with on-board processing.

### 2.1. Data Acquisition and Preprocessing

In this paper, we used two different data sets for ML model development as well as validation. The first one is a *control data set* acquired in an outdoor (i.e., field) environment with an effort to use optimal lighting and other imaging environmental settings. The second *"in-field" data set* is a more realistic one, collected in the field with soybean crops with diverse environmental variability. Details of these data sets are provided below along with the specific preprocessing steps used in this study.

#### 2.1.1. Control Data Set

**Figure 1:**
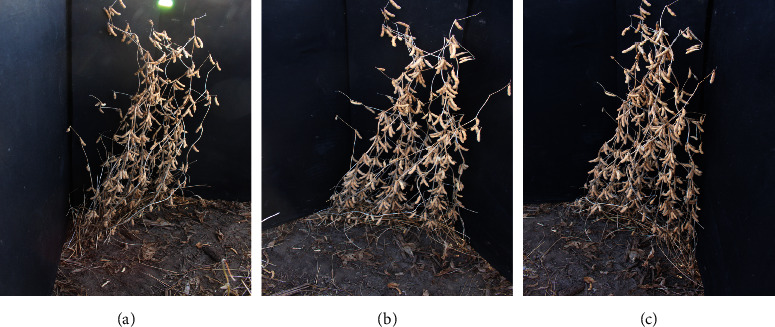
Sample images from the *control data set* along the background trifold used to remove background noise. These three views correspond to the same plot with multiple plants: (a) left view; (b) front view; (c) right view.

The images in this set were collected from random 30.5 cm subsections with soybean plants from matured soybean plots in 2014 (145 subsections) and 2015 (154 subsections). Three images were taken for each subsection using a trifold black background, which was used to remove background artifacts from the images as seen in Figure 1. Images were taken with a Canon EOS Rebel T5 in the RAW 18 megapixel format and were converted to jpg for processing. Pod sizes approximately range from 30 to 4000 pixels with an average size of 2000 pixels. The focus and white balance were set to auto and were adjusted as per the prevailing conditions. Upon the completion of imaging, plants from the 30.5 cm sections were cut from the ground level using a sharp clipper and bundled in a bag for pod counting. Care was taken to ensure no plant part loss occurred, enabling accurate pod counting. One subsection in 2015 had four images that were taken instead of three images, so we did not use information from this subsection.

**Figure 2:**
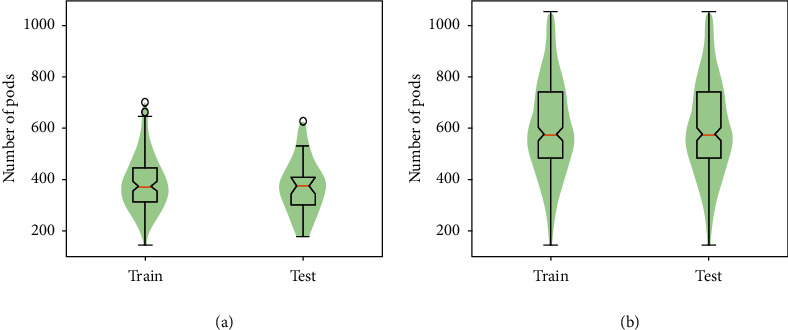
Pod count distributions for *control* and *in-field data sets*. (a) Pod count distribution for training and test subsets within the *control data set*. (b) Pod count distribution for training and test subsets within the *in-field data set*.

*Preprocessing*: expert raters labeled images for the 298 subsections using the VIA (VGG Image Annotator) image labeling software [21] to create the bounding boxes for pods. This data was split into two subsets for training (247 plots) and testing (51 plots) the ML models. Statistical characteristics of these data sets are provided in Table 1 and in Figure 2(a).

#### 2.1.2. In-field Data Set

**Figure 3:**
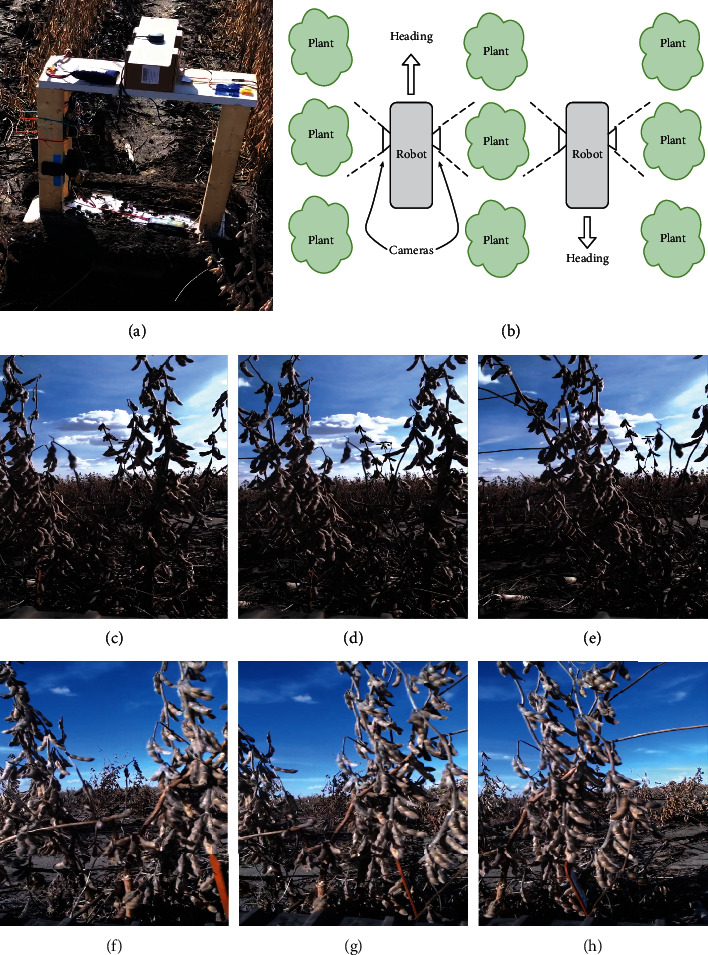
The top row (a, b) illustrates the ground robots' sensor setup and how it traverses the field, while images from (c) to (h) in the bottom two rows are typical video frame images, taken by the ground robot, viewing the same plot from the north and south sides. (a) Sensor setup on the ground robot. (b) Diagram demonstrating how the ground robot typically traverses a soybean field test. (c) North: image 1. (d) North: image 2. (e) North: image 3. (f) South: image 1. (g) South: image 2. (h) South: image 3.

This data set consisted of images taken from a ground robot [12]. The ground robot was outfitted with a wooden frame with a mounted webcam (Logitech C920) to capture images, ensuring that the full length of the soybean plants in each plot were imaged in each frame (Figure 3(a)). Videos were captured at a frame rate of 30 frames per second and at 720 p resolution. Pod sizes approximately range from 150 to 9000 pixels with an average size of 1200 pixels. The camera configuration permitted filming the two sides of the soybean plots with fewer passes (Figure 3(b)).

This data was collected from the USDA GRIN mini core collection [22] genotypes grown in a field near Ames, IA, in 15.24 cm length and 76.2 cm plot to plot distance. All plots were hand harvested at the R8 growth stage [23], after they were imaged with the ground robot. The plant height of genotypes from these plots ranged from 25 cm to 108 cm with a median height of 70 cm and a standard deviation of 15.99 cm. All plots were also rated for lodging on a scale of 1-5 with a score of 1 being all plants being erect and a score of 5 being prostrate. In this study, 46% of the plots were scored as a 1, 24% were scored as a 2, 13% were scored as a 3, 10% were scored as a 4, and 7% were scored as a 5. The genotypes varied in pod and pubescence colors, with 60% of the genotypes having a brown pod color and 40% having a tan pod color. 26% of the lines had light tawny pubescence, 32% had tawny pubescence, and 42% had gray pubescence. Genotypes could be further separated into elite, diverse, and plant introduction (PI) types representing commercial varieties to unimproved introductions [24]. The overall mean of the elite genotypes was 641 pods per plot (range of 313 to 1038 pods), diverse genotypes had a mean of 623 (range of 142 to 1058 pods), and PI had a mean of 466 pods (range of 150 to 805 pods).

*Preprocessing*: overall, we selected 123 plots in this data set. An expert rater determined the start and end of the frame sequences for each plot in each pass. This is to ensure that the frames accurately correspond to the plots for which manual pod counting was performed to obtain ground truth. The number of video frames per plot ranged from 13 to 98, with a median of 38 frames per plot. As we will consider only a few frames (1 to 3 in this study) per side of a plot to estimate yield, different sets of frames corresponding to a plot can be taken as different samples. We use this logic to perform data augmentation and form training and testing sets with 234 and 45 plot samples, respectively (i.e., total of 279 samples). The first step was to set aside 45 plots for testing, and the remaining 78 plots were used for training. The training set was augmented using different frames from these 78 plots. Each of the plots used in the training set was augmented 3 times giving a final set size of 234; no augmentation was performed in the testing set. Statistical characteristics of these data sets are provided in Table 1 and in Figure 2(b).

### 2.2. Machine Learning Framework for Yield Estimation

Soybean seed yield estimation through automated detection and counting of soybean pods is a challenging computer vision task. Complexity of this problem arises from various factors such as cluttered visual environment, occlusion of pods in a video frame, and lighting variations. One possible approach to address these issues at least in part is to consider multiple video frames for a plot from different viewing angles. Therefore, we propose a deep learning-based multiview image fusion framework that builds on a core model for pod detection and localization. In addition, to deploy this yield estimation framework on board a robotic platform, we need to detect and keep track of individual plots in real-time. In this regard, we also build a plot detection and tracking framework that can provide the necessary video frames for all plants in a specific plot to the yield estimation module. The proposed machine learning frameworks are described below.

#### 2.2.1. Pod Detection and Yield Estimation

Our pod count (as measurement of seed yield) estimation model takes multiple RGB images of the same plot, with multiple plants in commercial planting density, from different viewing angles. The key idea behind our proposed deep multiview image fusion framework is to build an end-to-end deep learning architecture that is able to ingest multiple image frames from different perspectives of a soybean plant and provide an overall estimate of pod counts for the plant. Our main hypothesis is that via such multiview image fusion, the model could learn to overcome pod occlusion problems, mitigate possible image quality issues encountered during the automated image selection process from videos (i.e., sequence of frames), and data heterogeneity encountered in real-life soybean experiment, breeding, and production fields. The model architecture is presented below.

**Figure 4:**
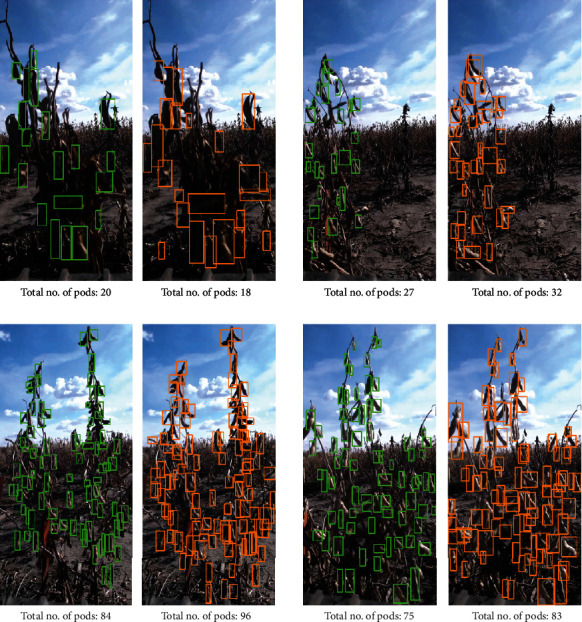
Plot samples with their respective annotated (left) and detected (right) pods. Detection IoU threshold set to 0.5. Four different examples here show the data diversity in the *in-field data set*.

*(1) Model Architecture and Training Process*. Our deep learning framework for multi-image fusion has two primary tasks: (i) pod detection on a soybean plot (with multiple plants) based on an individual image frame and (ii) estimating an overall pod count per plot based on multiple image frames. We choose a RetinaNet model architecture [25] with a VGG19 backbone to execute the first task. During the first phase of training, we trained the RetinaNet model to detect and isolate pods on soybean plants per plot as shown in Figure 4. For the *control data set*, 99 images were randomly selected and annotated using a single class “Pod” to train such a model. On the other hand, we use 513 randomly selected images from the *in-field data set* for training a RetinaNet model for pod detection in a field setting.

**Figure 5:**
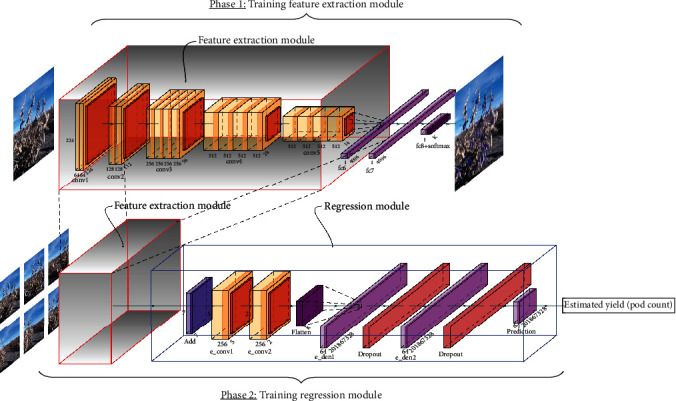
Approximate yield (pod count) estimation model architecture consisting of a feature extraction module (FM) and a regression module (RM) diagram.

After training and validation of a RetinaNet model, we focus on the next task of fusing information from multiple images to estimate the pod count for a soybean plot. The crux of the idea here is to leverage the features extracted by the pod detection model from the different images and map them to the overall pod count. To implement this, we use the lower 16 convolution layers of the VGG19 backbone of the trained RetinaNet model as the feature extractors (see [26] for the detailed structure of VGG19). We call this part of the model the feature extraction module (FM) as shown in Figure 5. The features extracted by the FM layers from multiple images are concatenated and are used as inputs in a regression module (RM). The RM has three consecutive convolution layers, with their respective max-pooling and batch normalization layers, except for the last convolution layer. These convolution layers are followed by a flatten layer and three fully connected layers as shown in Figure 5. The output of RM is the pod count for the plot consisting of multiple plants. We freeze the FM layers (taken from a well-trained RetinaNet model for pod detection) in order to train the RM layers. For the *control data set*, we started with only the front view image of a plot as the input (to FM) and then added two side views for the multiview version of the model. On the other hand, for the *in-field data set*, input images come in pairs, taken from the opposite sides by the robotic platform. We experiment with one image from each side (for the single view model) as well as with three images from each side (for the multiview model) of the plot. The training and test data distributions are already discussed in Section 2.1 and Table 1. All model variations were trained and validated using a PC workstation (OS: Ubuntu 18.04, CPU: Intel Xeon Silver 4108, GPU: Nvidia TITAN XP, RAM: 72 GB).

#### 2.2.2. Plant Detection and Tracking in Plots

In order to deploy the proposed yield estimation framework in an on-board, real-time fashion, we need to isolate the image frames corresponding to individual plots from the streaming video sequence collected by a camera on the robotic platform. However, in addition to isolating the image frames, if the plant area can be isolated in the image frame, then that part of the image can be used for pod detection. This can help in reducing the negative effects of other plants (from other neighboring plots) in the background as well as other background clutters. Therefore, we first focus on detection and isolation of plants from a plot in video frames.

*(1) Model Architecture and Training Process*. Similar to the pod detection model, we use a RetinaNet model with VGG19 backbone to detect and isolate the primary plot in an image frame. To train this model, we annotate about 1000 images from the *in-field data set* with diverse background conditions as well as diverse shapes and sizes of soybean plants. We use 90% of the annotated samples for training and the rest for validation.

Upon detection, we track the plants in a plot through the video frames with a unique ID tag such that the pod count estimation process can extract multiple frames for a specific plot and does not end up overcounting pods. There are two main aspects in a tracking algorithm. First, we detect and locate the targeted object in a frame, in our case, a soybean plot with multiple plants. Second, we decide whether the targeted object is present in subsequent frames. In our specific implementation, when the detector detects a plot, we save the information as central point of the bounding box. Each of the new central point is offered a unique ID and the location of those points is compared with the points in the subsequent frame, using Euclidean distance in a pair-wise manner. Based on the minimum distance, two central points (i.e., bounding boxes) are assigned to the same plot. If a new plot appears, the central point of that plot is isolated, and a new unique ID is assigned. If the current plot disappears or does not get detected, the corresponding central point is saved as an existing point. The ID is removed if the corresponding central point does not appear for several frames (five frames in our implementation).

## 3. Results

In this section, we present the performance of our proposed framework in the context of soybean pod detection and pod count estimation. We also evaluate the usefulness of our pod count or yield estimation outcomes in breeding practices. Finally, for applicability of these outcomes in varied plots and images to ensure utilization in field breeding, we show some anecdotal performance of our plant detection and tracking framework.

### 3.1. Pod Count Estimation Results

The pod detection model was validated using the Mean Average Precision (*m*AP) metric as given by Equation (1), by setting the Intersection over Union (IoU) threshold at 0.5. Details of *m*AP and IoU can be found in the supplementary material (available here). The *m*AP scores for the *control data set* and *in-field data set* were 0.59 and 0.71, respectively. However, it is important to note that the *control data set* had significantly lower pod annotations compared to the *in-field data set*. The rationale of having a smaller set of pod annotations in the *control data set* was that even with such a small training set, the overall pod count estimation performance was acceptable and is covered in more details in the following paragraph. Few anecdotal results for pod detection and isolation are presented in Figure 4.

**Figure 6:**
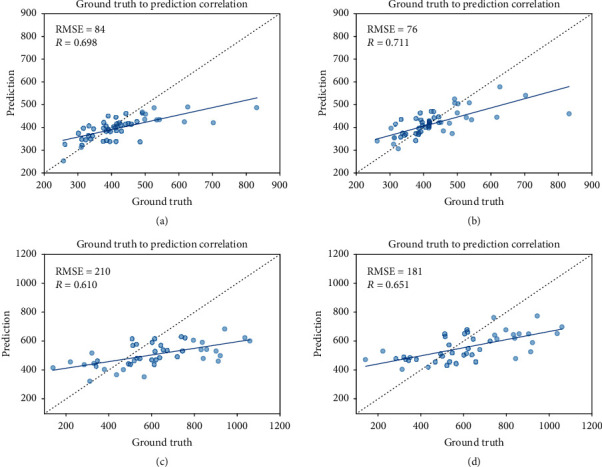
Correlations between ground truth and estimated pod counts using one and three images for the *control* and *in-field data sets*. (a) Correlation for the one-image input model with the *control data set*. (b) Correlation for the three-image input model with *control data set*. (c) Correlation for the one-image (per side) input model with the *in-field data set*. (d) Correlation for the three-image (per side) input model with the *in-field data set*.

The correlations between the ground truth and the predicted pod counts for both the *control data set* and the *in-field data set* are provided in Figure 6. We observed that fusing multiview images does help in improving the correlation between ground truth and prediction for both data sets. However, as expected, the performance is better for the *control data set* (Figures 6(a) and 6(b)), which can be attributed to the less occlusion and clean background with sharp color contrast with the foreground objects (plant and pods in this case). Interestingly, an improved pod count estimation performance for the *control data set* was noted, despite using a feature extraction module that shows a lower *m*AP value for pod detection and localization (due to smaller size of training data). Although, these moderate correlations may be acceptable for breeding purposes and applications, the predicted pod counts had narrower ranges compared to the ground truth pod count ranges. This can be attributed to the training data distribution shown in Figure 2 where most data points lie close to the mean value and we end up with unbalanced data sets with less representations from extreme pod count values.

### 3.2. Genotype Ranking Performance

**Figure 7:**
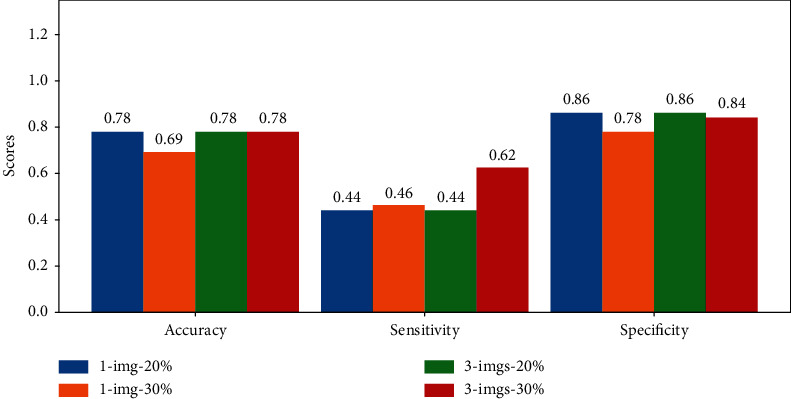
Ranking scores for the one- and three-image per side models on the *in-field* test data set.

While the correlation between pod count (i.e., proxy for yield estimation) prediction and the ground truth is an important metric for our proposed framework, from a breeding practice perspective, it is also important to make sure that the yield estimation framework is useful to downselect the top performing genotypes. For example, a 30% selection cut-off means that only those plots that rank among the top 30% (in terms of yield) are selected to advance to the next generation and subsequent testing (next season or year) in the breeding program. In this study, we use both 20% and 30% selection criteria to validate our framework, as these are reasonable downselect (i.e., culling) levels in a breeding program. Standard ML metrics such as accuracy, sensitivity, and specificity are presented for evaluation (Figure 7 and Table 2). However, as our test data set sizes are rather small (51 test samples for the *control data set* and 45 test samples for the *in-field data set*), we also provide the actual numbers of true positive, true negative, false positive, and false negative samples in Table 2.

### 3.3. Plot Detection and Tracking Performance

**Figure 8:**
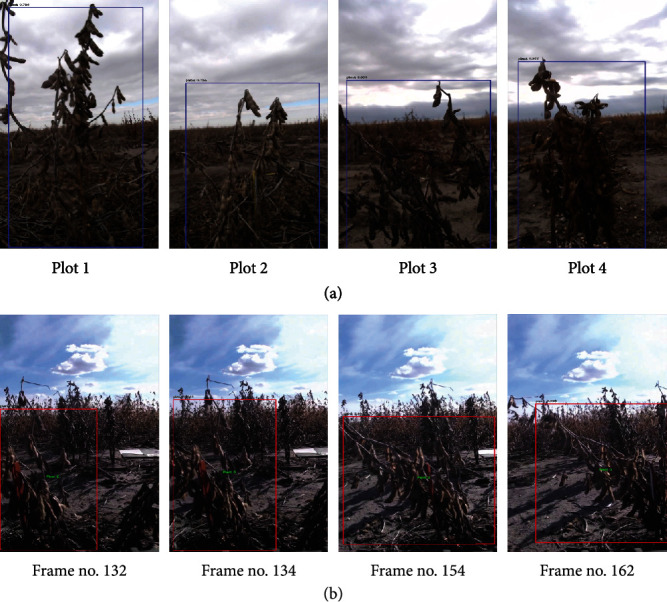
Samples of plot detection and tracking from recorded videos by the ground robot system. (a) Samples of plots detected while the robot travels on the same row. (b) Sequences of image frames while detecting and tracking a single soybean plot from an input video.

We provide few results of our plot detection and tracking models in Figure 8. We report that plants of various sizes and shapes can be detected and sufficiently isolated despite a cluttered background with very low contrast. Although in practice we see that our plot detection and tracking system is mostly reliable, performance can suffer in low-light conditions as well as in severe occlusion scenarios, specifically due to large and lodged (nonupright) plants. Our future work will go beyond this anecdotal study to generate statistically significant quantitative results for a fully end-to-end on-board real-time soybean yield estimation system. However, we clearly show the feasibility of such a system in this paper.

## 4. Discussion

From the results, it is clear that performance for the *control data set* is slightly better compared to that for the *in-field data set*, which conforms to our earlier correlation results, and is also true from the domain experience. However, in this particular data set, we only see a marginal improvement in performance with the usage of multiview images as opposed to only single view images. Still, for the *in-field data set*, we noted that single view images from both sides of the plots are still necessary (shown in Figures 3(c)–3(h)). Overall, our results show that the proposed framework could be quite useful for selecting top performing genotypes especially when using a 30% selection criterion compared to a 20% selection criterion as evidenced with a higher sensitivity score. However, if a plant breeder is more interested in discarding the bottom performers, they can achieve reasonable success at the 20% selection level too due to high specificity and lower sensitivity scores. This is particularly of importance in early stages of yield testing, where breeders are more concerned about “discarding” unworthy entries rather than “selecting” the top performers as the tests do not have sufficient statistical power to separate mean performance corresponding to the phenotypic and breeding values. With an improvement in test data size, it is possible that model performance (i.e., sensitivity) may also improve enabling high confidence using more stringent downselection or culling levels. With small test data sets, it is difficult to draw strong conclusions in such a discrete classification setting (where even one or two samples can change the overall statistics). Therefore, future work will focus on substantially increasing the test data set size to arrive at statistically more significant conclusions.

While similar deep learning models have been studied extensively for organ detection in fruit trees, pod detection, counting, and an overall yield estimation from a ground robotic platform present a harder challenge due to issues such as the increased level of occlusion, limited camera views, and cluttered backgrounds. Our experiments showed promising results in a controlled outdoor environment; although model performance was lower for the outdoor *in-field* data set, the results are still quite encouraging. We attribute the degradation of the model prediction to the lower quality of the data we had from the *in-field data set* in comparison to the *control data set*. The bottleneck of our experiments was not the ability to image plots, but the time and effort it took to manually count pods for every plot at a very high level of fidelity. One of the benefits of this type of yield estimation is that it focuses on physically quantifying every pod in a plot, which has a direct correlation with the yield. We observed a correlation of 0.87 and 0.90 between manual pod count and seed yield for the *in-field* and *control sets*, respectively. Averages of 2.0 and 2.2 seed/pod were noted for the *in-field* and *control set*, respectively.

Due to the variability in the breeding program layout and pipelines, we suggest a balanced and strategic approach of using small weight autonomous ground robots in early generation, progeny row, and preliminary and unreplicated advanced replicated yield trial stages, where genotypes can be grown in multiple locations but only one location needs to be harvested to obtain the seed source for the next season planting. Additionally, in replicated tests, only one replication can be harvested for the seed source, accruing additional resource savings. Furthermore, plant breeding programs are also at the mercy of weather events. For example, excessive rainfall in the fall season in North American soybean growing regions often complicates machine harvest, bringing the entire program to a halt causing significant delay in data analysis and advancement decisions for winter nursery operations or for the next season. Also, breeding decisions for making selections and advancements need to wait for machine harvest data, delaying breeding cycles and turnaround times. The deep multiview image fusion architecture can empower breeding programs to operate in wet soil conditions where machine harvest is not possible. In all these above scenarios, except for the harvest plot, all other plots will be imaged using the ground robot and the yield estimated using the application of ML models by obtaining information on plant reproductive organs (e.g., pods) of economic importance, for seed yield estimation.

To provide a context of the time and resource investment, 50-100 soybean yield plots can be combined in one hour at each testing field site depending on plot sizes and number of rows, etc., by a 1-2-person work crew. Most breeding programs harvest in the tens of thousands of plots in a given year. However, a mobile ground robot can image up to 300 plots per hour. This shows the exponential advantage of the approach presented in this paper over traditional methods of harvesting plots for yield data. Ultimately, these situations and the integration of yield estimation in breeding methods and pipelines using ground robots can remove the need to harvest all plots from all locations, to save time and resources as described earlier.

We continue to deploy different ML-based methods for trait phenotpying including the root nodule count Soybean Nodule Acquisition Pipeline (SNAP) that quantifies a nodule by combining RetinaNet and UNet deep learning architectures for object (i.e., nodule) detection and segmentation [27], microscopic nematode egg detection in cluttered images [28], and root trait phenotyping [29, 30]. Therefore, advances from other problem definitions can cross-inform and improve object detection models. While we are integrating deep multiview image fusion in our breeding program, our current work focuses on a few major areas. First, we are exploring to improve the image gathering quality using an online feedback control system that interacts with the robot to navigate the fields in an automated manner. Second, we are developing algorithms capable of matching video frames to accurate plant locations and determining the best frames to use for a plot. Since both data sets were relatively small in size compared to many other use cases of deep learning frameworks, we are increasing the data set size for further model improvements, similar to other existing nonsoybean data sets such as the Global Wheat Head Detection data set [31], and the MinneApple data set [32]. We also note that active learning algorithms will also be useful to reduce the amount of labeling needed by deep learning models to achieve good predictive performance [33].

## 5. Conclusion and Future Work

ML and more specifically DL methods continue to open previously inconceivable phenotyping doors for breeding and research application [34–36]. In this paper, we propose a deep multiview image fusion architecture capable of reducing overhead in the yield testing trial. This was achieved with minimum human intervention by properly estimating yield, using pod count estimation, and ranking soybean genotypes for making breeding decisions. Our proposed method uses a deep learning framework that performs soybean pod detection and yield estimation using single or (fusing) multiple RGB images of a plant collected from a mobile motorized ground phenotyping unit. Although pod estimation is a proxy or surrogate trait for yield estimation, it is one of the important yield components of overall seed yield [37]. Most other yield prediction methodologies in plant science have focused on above canopy measurements such as reflectance measurements [8, 9] and canopy coverage [38]. Future work should focus on the above problems, as well as moving integration of automated ways to obtaining yield component traits with indirect estimation of physiological traits and indices. These can be deployed at all field and controlled environment growing conditions, allowing for larger sampling sizes without the need for labor-intensive pod counting tasks and/or machine harvest of all plots. If high fidelity rankings can be achieved in full breeding plot tests, this methodology could help to greatly reduce the labor and time required for harvest operations in a breeding program in any given year. This will allow for less issues related to timely harvesting of plots, as well as faster decision making in a breeding program, with data available to a breeder sooner than would typically be available with traditional harvest methods. The utilization of this pipeline with drone-based phenotyping is very exciting [39]. Ultimately, this deep multiview image fusion framework can be deployed in a breeding pipeline to improve the capability to obtain high quality seed yield data without the need to machine harvest all plots, one of the most time- and resource-intensive steps in plant breeding.

## Figures and Tables

**Table 1:** Descriptive statistics of the data sets include the *control* and *in-field data sets*.

Data sets	Control set		In-field set	
	Train	Test	Train	Test
No. plots annotated	247	51	234	45
Number of pods				
Minimum	144	257	150	142
Maximum	704	831	1038	1058
Mean	396.2	423.9	595.3	611.5
Standard deviation	99.89	106.61	182.11	219.75

**Table 2:** Model-predicted ranking results for the top 20% and 30% plots from the *in-field data set* using one and three images (per side) for pod counting.

Ranking	1 img: control set		3 imgs: control set		1 img: in-field set		3 imgs: in-field set	
	Top 20%	Top 30%	Top 20%	Top 30%	Top 20%	Top 30%	Top 20%	Top 30%
True positive	7	12	7	10	4	6	4	8
True negative	37	33	37	31	31	25	31	27
False positive	3	3	3	5	5	7	5	5
False negative	4	3	4	5	5	7	5	5
Accuracy	0.86	0.88	0.86	0.80	0.78	0.69	0.78	0.78
Sensitivity	0.64	0.80	0.64	0.67	0.44	0.46	0.44	0.62
Specificity	0.93	0.92	0.93	0.86	0.86	0.78	0.86	0.84

## Data Availability

Data and codes will be made available publicly after acceptance of the paper through corresponding authors' GitHub pages.
